# Diligent for better or worse: Conscientiousness is associated with higher likelihood of suicidal behavior and more severe suicidal intent in later life

**DOI:** 10.1016/j.comppsych.2024.152523

**Published:** 2024-08-06

**Authors:** Anna Szücs, Hanga Galfalvy, Maria G. Alessi, Laura B. Kenneally, Jose M. Valderas, Andrea B. Maier, Katalin Szanto

**Affiliations:** a Vrije Universiteit Amsterdam, Faculty of Behavioural and Movement Sciences, The Netherlands; b National University of Singapore, Yong Loo Lin School of Medicine, Department of Medicine, Singapore; c Columbia University, Department of Psychiatry, USA; d University of North Carolina at Charlotte, Program in Health Psychology, USA; e Virginia Consortium Program in Clinical Psychology, USA; f University of Pittsburgh, School of Medicine, Department of Psychiatry, USA

**Keywords:** Suicidal behavior, Depression, Personality, Conscientiousness, Aging, Physical illness

## Abstract

**Background:**

Contradictory findings link trait conscientiousness in mid- and late life to increased healthspan and lifespan, as well as to death by suicide. It remains unclear whether conscientiousness is associated with higher odds of attempting suicide or with more severe suicidal behavior among attempters, and whether its relationship to suicide risk varies with aging-related stressors, such as declining health.

**Methods:**

In this cross-sectional study comprising 313 depressed adults aged ≥40 years and participating in the Longitudinal Research Program in Late-Life Suicide (Pittsburgh, USA), we employed logistic and linear regression to test whether conscientiousness was associated with the presence of recent suicidal behavior (≤2 years) and with intent severity in recent attempters (*n* = 84). We further tested whether the above relationships varied based on mental, cognitive, and physical health status, measured as depression severity, cognitive functioning, and the presence/absence of severe physical illness.

**Results:**

Participants were 62.1 years old on average (SD = 7.6), 85% White, and 53% female. Recent attempters had a mean age of 61.8 years at their most recent attempt (SD = 8.5), had lower cognitive functioning and were more likely severely physically ill than comparisons. Conscientiousness was positively associated with a higher likelihood of recent suicidal behavior overall (adjusted OR = 1.44, 95% CI = 1.09, 1.90, *p* = .010), but not in case of co-occurring severe physical illness (interaction OR = 0.54, 95% CI = 0.30, 0.97, *p* = .039). Conscientiousness was also positively associated with suicidal intent at the most recent attempt (adjusted β = 1.60, SE = 0.62, *p* = .012), explaining 7% of its variance, although this association lost significance after adjusting for other personality dimensions.

**Conclusions:**

Highly conscientious middle-aged and older adults may be at increased risk of resolute suicidal behavior, although conscientiousness may not confer additional suicide risk among those severely physically ill.

## 1. Introduction

Suicide in mid- and late life is a serious public health concern worldwide. In the United States, adults over age 85 years have the highest rate of death by suicide of all age groups [1]. From 2021 to 2022, the suicide rate of adults aged 65+ years demonstrated an increase of 8.1%, the greatest change of all age groups, second to those aged 45–64 years at 6.1% [1]. Whereas older adults make fewer suicide attempts than younger adults, they are more likely to die as a result of their first attempt [2]. Consequently, suicidal behavior history, considered the strongest predictor of suicide risk in younger populations [3], loses its predictive value with age [4]. Given the higher lethality of suicidal behavior in middle-aged and older adults, research identifying predictors of suicide risk specific to this population is needed.

Declining health is one of the most common stressors during aging. Mental, cognitive, and physical health decline have been independently associated with suicidal behavior in older adults [5–7]. However, most middle-aged and older individuals with physical, cognitive, or mental health issues never attempt or die by suicide, making these risk factors non-specific for suicidal behavior. Declining health likely affects aging adults differently, based on their individual characteristics.

In line with the stress-diathesis model widely used in suicide research [5], specific stressors such as declining health could trigger suicide risk through their interplay with certain constitutional traits. In a Norwegian qualitative study, informants described older suicide decedents as action-oriented and controlling [8] and considered that their suicidal act possibly motivated by their fear of aging-related functional decline [9]. Among personality traits commonly used in quantitative research, conscientiousness may map best on action-orientedness and need for control, based on its definition of being goal driven, dependable and meticulous, as opposed to easy-going, careless, and disorganized [10].

Although studies conducted in adults of all ages reported either negative or no associations between conscientiousness and suicide risk [11,12], studies focusing on adults aged 50+ [13,14] and 65+ years [15] highlighted notable positive associations. Conscientiousness was higher in suicide decedents than in suicide attempters [13,15] and orderliness, a conscientiousness subdimension, was higher in suicide attempters with their first attempt after age 50 than in depressed non-suicidal comparisons and depressed non-attempters with active suicidal ideation [14]. Yet, in these studies, older suicide attempters and decedents had lower or similar levels of conscientiousness and orderliness relative to non-psychiatric comparisons [14,15], suggesting that they may only be more conscientious than other depressed individuals but not more conscientious than the general population.

This is further supported by the well-established positive associations of conscientiousness with health and healthy aging. Conscientiousness has been linked to lower disability, morbidity, and mortality [16,17], and to higher perceived control during the aging process [18]. It is positively associated with various adaptive outcomes, such as healthier day-to-day lifestyle choices [19,20] and higher treatment adherence [21], even for particularly demanding treatment regimens [22,23]. However, some prospective findings among smokers described positive associations with smoking overall, and between the self-discipline subdimension of conscientiousness and smoking in response to daily hassles [19], setting precedent for associations between conscientiousness and maladaptive behavior in stressful circumstances.

Conscientiousness can indeed take maladaptive forms manifesting in excessive need for control [24]. Expression of negative emotions, in both controlled and uncontrolled ways, is rarely employed as a coping strategy by highly conscientious individuals [25], which can make coping with long-term distress difficult. It may be possible that highly conscientious aging adults facing depression or other forms of uncontrollable health problems may attempt to regain control in maladaptive ways, for instance by resorting to suicidal behavior. Nonetheless, extant findings about the direct effect of conscientiousness on suicide risk remain inconclusive [26] and research on interactions between personality traits and aging-related stressors is generally scarce [27,28]. Confirming potential links between conscientiousness and suicide risk appears an important first step to detect individuals vulnerable to aging-related stressors early on.

This cross-sectional study aims to answer the following research questions: Is conscientiousness positively associated with higher likelihood (Q1a) and intent severity (Q1b) of suicidal behavior in middle-aged and older adults with unipolar depression? If present, are these associations robust to adjustment for other individual characteristics (Q2a-b)? Finally, are these associations amplified in those who have poorer health status in core domains of healthy aging, namely mental, cognitive, or physical health [29] (Q3)?

## 2. Methods

### 2.1. Participants

The sample consisted of 313 unipolar depressed adults aged 40 years or older participating in a larger ongoing longitudinal study, the Longitudinal Research Program in Late-Life Suicide at the University of Pittsburgh [30]. Included participants were at least 40 years old and reported clinically significant depressive symptoms, indicated by a score of 14 or greater on the 17-item *Hamilton Rating Scale for Depression* [31]. Participants were ineligible for the study if they scored lower than 23 on the *Mini Mental Status Exam* [32], or had a documented history of major neurocognitive disorder/impairment. Participants were also excluded for any history of stroke, neurological disorder, psychotic disorder, major depression with psychotic features, mania/hypomania, or electroconvulsive therapy within six months of study entry. Lifetime psychotic disorder or mania was assessed using the *Structured Clinical Interview for DSM-IV* [33]. Participants were enrolled in the study as depressed non-attempters if they had no prior history of suicidal behavior or as depressed suicide attempters if they had ever enacted self-injurious behavior with the intent to die [34] and had suicidal ideation with a plan at the time of consent based on their score on the *Beck’s Scale for Suicidal Ideation* within one month [35]. All inclusion/exclusion criteria, including attempt history, were determined by consensus of all available information, including participant report, medical records, and informant report when available. Participants were excluded if there was notable discrepancy across information sources. The present cross-sectional study focused on recent suicidal behavior as an outcome, defined as an attempt within the last two years, to ensure analyses captured later-life behavior (see Analytic Approach). Data availability defined the size of the sample, which included all depressed participants who had filled out the *NEO Five-Factor Inventory* [36], employed to measure conscientiousness (see Assessments below).

### 2.2. Assessments

#### 2.2.1. Sociodemographics

Participants’ age, sex, race, years of education, marital status, and per capita yearly income, were collected upon enrollment. Marital status was missing in one participant, and yearly per capita income was missing in 16 participants. These variables were only used for sample characterization (see “Analytic approach”).

#### 2.2.2. NEO Five-Factor Inventory (NEO-FFI) – conscientiousness

The 60-item *NEO Five-Factor Inventory* [36] was administered as a self-report, with each item assessed on a 5-point Likert scale (0 = strongly disagree to 4 = strongly agree). The NEO-FFI comprises five 12-item subscales, each assessing a different ‘Big Five’ personality dimension, namely neuroticism, extraversion, openness to experience, agreeableness, and conscientiousness. Our study intended to test a priori hypotheses regarding conscientiousness, hence we only employed the other subscales in sensitivity analyses (see “Analytic approach”). Regarding missingness on the NEO-FFI, three participants discontinued completion before the end, resulting in missing three to four items on each subscale (three conscientiousness items). Openness had an additional missing observation due to a skipped item by one participant. Subscale scores were therefore calculated as the mean of the completed items, rather than as a sum score.

#### 2.2.3. Hamilton Rating Scale for Depression (HRSD)

The clinician-rated 17-item *Hamilton Rating Scale for Depression* (HRSD) was administered to assess severity of current depressive symptoms at time of consent [31]. The suicidal ideation item (Item 3) was excluded from total scores in analyses given that the study’s outcomes also measured suicide risk. Higher scores on the HRSD indicate more severe depression.

#### 2.2.4. Dementia Rating Scale (DRS)

The total score from the *Mattis Dementia Rating Scale* (DRS) characterized global cognitive ability [37]. Higher scores indicate better cognitive functioning. The DRS was missing in 31 participants. This missingness occurred because the DRS was part of a larger cognitive test battery completed at a different visit than the rest of the baseline assessments. The data was established to be missing completely at random by Little’s test (χ^2^ = 63, df = 53, *p* = .163). To address missingness, we employed multiple imputation using R’s mice package [38] set to generate 10 imputed datasets with 10 iterations each.

#### 2.2.5. Cumulative Illness Rating Scale - Geriatric (CIRS-G)

Physical illness burden was measured using the clinician-administered *Cumulative Illness Rating Scale for Geriatrics* (CIRS-G; [39]). All diagnosed physical conditions, excluding psychiatric illness and any medical consequence of an attempt, were rated by organ system and illness severity on a scale from 0 (no problem) to 4 (extremely severe) to reflect increased morbidity. As has been done previously [40], overall illness burden was coded dichotomously in analyses to focus on illnesses life-threatening/impairing enough to act as a potential suicidal trigger. Severe physical illness was indicated by the presence, in any category, of a rating of ‘3’ (indicating chronic disability or illness not fully compensated for by first line medical treatment) or ‘4’ (extremely severe illness in late disease stage that required immediate treatment and associated with severe functional impairment).

#### 2.2.6. Characterization of suicidal behavior

Participants were asked about their total number of attempts. The *Beck Lethality Scale* [41] was used to characterize attempts’ medical seriousness.

Suicidal intent severity of attempts, one of the primary outcomes, was assessed using *Beck’s Suicidal Intent Scale* [42]. This scale gauges anticipated lethality of suicide attempt via 20 items, each scored between 0 and 2, assessing the likelihood of intervention due to objective circumstances, extent of preparations for the attempt, and expectations of fatality. Total scores are calculated as a sum, with higher scores indicating greater intent.

### 2.3. Procedure

All procedures were approved by the University of Pittsburgh’s Institutional Review Board (Protocol IRB0407166). Participants were recruited from psychiatric inpatient units, a geriatric intensive outpatient group, depression clinics, and from University of Pittsburgh research registries.

After giving written, informed consent, participants completed clinician-administered assessments and self-report questionnaires. At participants’ discretion, assessments were conducted in-person or online via software compliant with the Health Insurance Portability and Accountability Act (HIPAA). All study clinicians were required to demonstrate reliability of ratings with study psychiatrists before conducting clinical interviews.

### 2.4. Analytic approach

We tested associations between conscientiousness and recent suicidal behavior’s presence and intent severity. Recent suicidal behavior was defined as any suicide attempt occurring within the past two years. If more than one attempt occurred within the last two years, we considered intent severity of the most recent. We chose the two-year window to define recent suicidal behavior based on the proposed DSM-5 criteria for suicidal behavior disorder [43] and the association of this timeframe with an acute risk of re-attempting in multiple studies [44–46]. Employing binomial logistic regression (function *glm*, package stats), we evaluated the effect of conscientiousness on the presence of recent suicidal behavior (none vs. any attempt in the past two years) (Q1a-3a). Using linear regression (function *lm*, package stats), we tested the association between conscientiousness and severity of suicidal intent at the most recent attempt in the subsample with recent suicidal behavior (*n* = 85; Q1b-3b).

We built a set of three models for each of the above outcomes: single predictor models testing Q1a-b with conscientiousness as single independent variable; covariate-adjusted models testing Q2a-b additionally including age, sex, and the three health domains (depression severity, cognitive functioning, and the presence of severe physical illness); and interaction models testing Q3a-b, where the three health domains were entered in interaction with conscientiousness. In case of any significant interaction, a follow-up model deconstructed it into dummy-coded levels to facilitate interpretability. Continuous variables were dichotomized by median split to enable dummy-coding (median = 3.42). The follow-up model contained no covariates.

To account for other potential confounders, we performed a sensitivity analysis testing the main findings’ robustness to additional variables differing between recent attempters and the comparison group (years of education; Table 1) and to the four other Five Factor dimensions, all of which significantly correlated with conscientiousness (Supplemental Fig. S2).

Multicollinearity was low, with variance inflation factors obtained from models with non-imputed data remaining below 2 for covariate-adjusted models, and below 2.6 for interaction models.

## 3. Results

### 3.1. Sample characteristics

Table 1 shows participants’ characteristics (*N* = 313) and comparisons between recent attempters (*n* = 84) and other participants (*n* = 229). The sample was aged 62.1 years on average (SD = 7.6), had a balanced representation of females and males (53.4 vs. 46.7%), and primarily identified as White (84.7%). Conscientiousness had a mean item score of 3.4 points (SD = 0.7; NEO-FFI item score range: 1–5 points), correlated negatively with neuroticism, and positively with extraversion, openness to experience, and agreeableness (Supplemental Fig. S2).

Recent attempters had 1.4 fewer years of education than the comparison group (13.9 vs. 15.3 years), higher levels of conscientiousness (3.6 vs. 3.4 points) and extraversion (3.0 vs. 2.8 points), and lower levels of openness to experience (3.1 vs. 3.2 points). Recent attempters had lower cognitive functioning than the comparison groups (mean DRS 134.0 vs. 136.3 points), and a larger proportion were severely physically ill, as defined by a score of 3 or higher for any physiological system on the CIRS-G (41.7% vs. 26.2%). All but one of the recent attempters reported some level of current suicidal ideation (98.81%), with a mean ideation score of 24.1 (SD = 7.3) on the SSI. By contrast, suicidal ideation was present in 62.9% of the comparison group, where those ideating had a mean SSI score of 13.9 (SD = 8.2). At the time of their most recent attempt, the average age of the recent attempters was 61.8 years old (SD = 8.5).

### 3.2. Association between conscientiousness and the presence and severity of recent suicidal behavior

(Q1a-b) In single predictor models (Table 2 first column), conscientiousness was associated with a higher likelihood of having had a recent suicide attempt (OR = 1.41; 95% CI = 1.09, 1.84, *p* = .010) and, among recent attempters, with higher suicidal intent severity of the most recent attempt (*β* = 1.40, SE = 0.58, *p* = .018). Conscientiousness explained 7% of the variance of suicidal intent (*R*^2^ = 0.07, *F*(1,82) = 5.81, *p* = .018).

(Q2a-b) In covariate-adjusted models controlling for age, sex, and health domains, namely depression severity, cognitive functioning, and severe physical illness (Table 2, second column), conscientiousness remained significantly associated with greater likelihood of recent suicidal behavior (OR = 1.44, 95% CI = 1.09, 1.90. *p* = .010) and with higher intent severity among recent attempters (*β* = 1.60, SE = 0.62, *p* = .012).

Sensitivity analyses tested whether the relationships between conscientiousness and the presence and severity of recent suicidal behavior were robust to years of education, a potential confound given its difference between recent attempters and comparisons (Table 1) and to other Five Factor personality dimensions (Supplemental Table S1). The positive relationship between conscientiousness and the presence of recent suicidal behavior was maintained after adjusting for years of education (OR = 1.44, 95% CI = 1.10, 1.90. *p* = .008), personality dimensions (OR = 1.47, 95% CI = 1.08, 2.04. *p* = .017), and all of these variables together (OR = 1.48, 95% CI = 1.08, 2.06. *p* = .017). The relationship between conscientiousness and intent severity was robust to adjusting for years of education (*β* = 1.21, SE = 0.55, *p* = .031), but not to personality dimensions (*β* = 1.03, SE = 0.72, *p* = .159), of which agreeableness was negatively associated with intent severity (*β* = −1.35, SE = 0.56, *p* = .018).

### 3.3. Health-dependent variation in the relationship of conscientiousness with the presence and severity of recent suicidal behavior

**Fig. 1.**
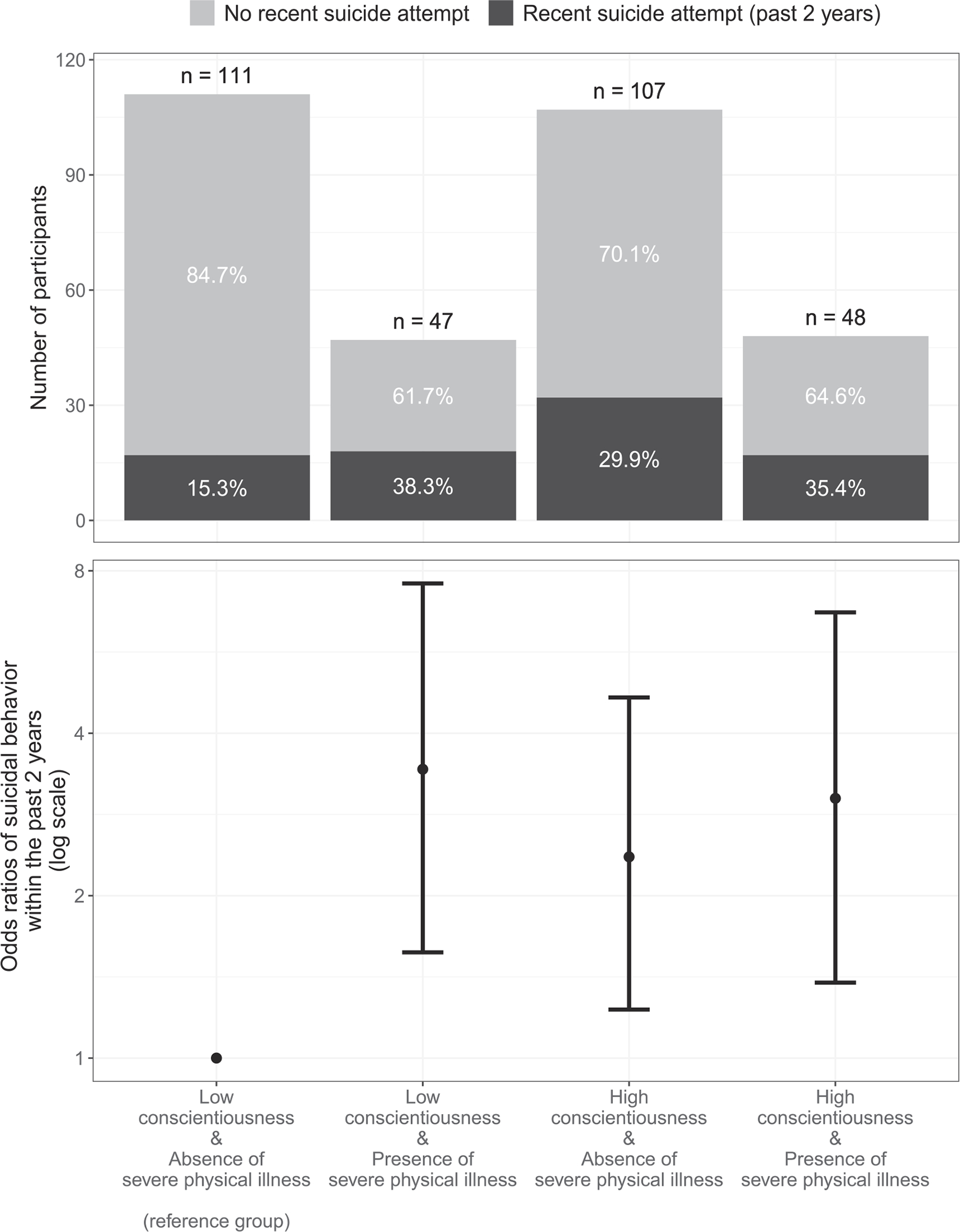
Interaction effect between conscientiousness and severe physical illness on the odds of suicidal behavior within the past two years decomposed into pairs of low-high conscientiousness and absence-presence of severe physical illness. Note: Participant counts and percentages for each pair of conditions are presented in the upper panel; odds ratios (points) and 95% confidence intervals (whiskers) are presented in the lower panel based on the regression model summary reported in Table 3, with “low conscientiousness & absence of severe physical illness” being the reference group set at an odds ratio of 1.

Of the three health domains (Table 2, second column), lower cognitive functioning (OR = 0.68, 95% CI = 0.52, 0.89, *p* = .005) and the presence of severe physical illness (OR = 1.91, 95% CI = 1.11, 3.30, *p* = .020) were independently associated with a higher likelihood of recent suicidal behavior, whereas depression severity had no association. None of the domains were associated with intent severity at the most recent attempt.

(Q3a) In the interaction model (Table 2, upper, third column), the relationship between conscientiousness and the presence of recent suicidal behavior was further qualified by the presence of severe physical illness (OR = 0.54, 95% CI = 0.30, 0.97, *p* = .039). A follow-up model where this interaction was dummy-coded for interpretability (Table 3) indicated that participants with low conscientiousness and no severe physical illness were less likely to have made a recent suicide attempt than their counterparts who were severely physically ill (OR = 3.43, 95% CI = 1.57, 7.58, *p* = .002), had high conscientiousness (OR = 2.36, 95% CI = 1.23, 4.66, *p* = .011), or both of these conditions (OR = 3.03, 95% CI = 1.38, 6.70, *p* = .006). Having both severe physical illness and high conscientiousness did not result in a higher likelihood of recent suicidal behavior than having either of the conditions separately (Table 3, Fig. 1). The relationship between conscientiousness and presence of recent suicidal behavior did not vary as a function of depression severity, or cognitive impairment.

(Q3b) The relationship between conscientiousness and intent severity at the most recent attempt did not vary as a function of health in any domain (Table 2, lower, third column).

In sensitivity analyses adjusting for years of education and other personality dimensions (entered in interaction with severe physical illness in individual models; Supplemental Table S2), a negative conscientiousness-by-severe physical illness effect predicting the presence of recent suicidal behavior persisted after adjusting for years of education (OR = 0.53, 95% CI = 0.30, 0.94, *p* = .030), openness to experience (OR = 0.49, 95% CI = 0.27, 0.88, *p* = .018), and agreeableness (OR = 0.54, 95% CI = 0.30, 0.95, *p* = .032). In models adjusting for neuroticism and extraversion, the conscientiousness-by-severe physical illness effect did not reach significance (resp. OR = 0.62, 95% CI = 0.33, 1.15, *p* = .129 and OR = 0.59, 95% CI = 0.32, 1.09, *p* = .091).

## 4. Discussion

We conducted a cross-sectional study in middle-aged and older depressed adults testing the direct and health status-dependent effects of conscientiousness on the likelihood and intent severity of recent suicidal behavior. We found that – independent of age, sex, and level of impairment in mental, cognitive, and physical health domains – more conscientious individuals were more likely to have engaged in recent suicidal behavior, and those who did had higher levels of intent at their most recent attempt. Yet, the likelihood of recent suicidal behavior was not associated with conscientiousness in individuals who were severely physically ill. Whereas the direct association between conscientiousness and the presence of recent suicidal behavior was unaffected by adjusting for the four other personality dimensions, this was not the case for its association with intent severity.

Our findings indicate that, in subpopulations of depressed middle-aged and older adults, conscientiousness may not only contribute to the risk of suicidal behavior but also to higher levels of determination and planning preceding an attempt. Conscientiousness is related to intolerance of ambiguity (need for closure) and personality rigidity, a form of dispositional resistance to change [47,48]. Thus, highly conscientious depressed adults may be more inclined to make radical decisions in the face of uncertain situations, and may have difficulty disengaging from these decisions afterward. These results corroborate our previous finding associating higher levels of orderliness – a conscientiousness subdimension – to suicidal behavior occurring after 50 years of age [14]. They also align with a Norwegian qualitative psychological autopsy study, where informants described suicide decedents 65+ years as action-oriented achievers; a profile consistent with high conscientiousness [8].

However, the same study also reported functional decline in different health domains as an important precipitant of the decedents’ suicidal act [9]. Our findings indicated no higher risk resulting from the combination of high conscientiousness and severe physical illness even though both factors were associated with recent suicidal behavior independently. This may suggest a plateau effect, namely, that suicide risk will not increase beyond a certain point even as risk factors accumulate. It could also be possible that conscientious traits, such as being goal-driven and meticulous, play a role in the way individuals manage their physical health, and foster a greater sense of control over severe physical illness. The belief that one has control over events in one’s life (internal locus of control) has been positively associated with conscientiousness [49], and conscientiousness also related positively to the management of severe chronic conditions such as HIV or end-stage renal disease [22,50]. Thus, conscientiousness may play a protective or an exacerbating role in the suicidal diathesis depending on context.

Further, the role of conscientiousness may partly overlap with other personality dimensions, as seen in our sensitivity analysis with low agreeableness (defined as antagonism, callousness) having a stronger association with suicidal intent severity. As the co-occurrence of the two traits is consistent with qualitative descriptions of older attempters [8], investigating combinations of personality traits may help further understand at-risk profiles for late-life suicidal behavior, as it has been done for other aging-related outcomes, such as survival in old age [51].

The present study offers a nuanced investigation of the effect of conscientiousness on suicidal behavior in mid- and late life. The well-characterized sample, hypothesis-driven approach, and careful sensitivity analyses serve as its strengths. The CIRS-G represents a more objective assessment of physical illness and functional limitations than do self-reports of subjective physical health, which are more highly intercorrelated with depression [52].

As this study relies on cross-sectional data, it could not inform causality or progression of suicide risk over time. Despite restricting recent suicidal behavior to the last two years, these results cannot control for recall bias, or the potential for the most recent attempt to have occurred before the onset of severe physical illness. Of note, temporality was less of a concern for conscientiousness, which has been identified as one of the most stable traits across the lifespan [53]. As our hypotheses focused on conscientiousness, we only investigated the other four personality traits as potential confounds. Finally, suicide attempt is only a proxy for suicide, further limiting generalizability of our work to actual suicide risk.

These limitations warrant longitudinal studies on the effect of conscientiousness and other personality traits on mid- and late-life suicidal behavior. Although the interplay between aging-related functional decline and personality has been studied in the general population [16–18], prospective data is lacking in vulnerable subpopulations. Future studies should also contextualize the effect of conscientiousness within constellations of personality dimensions and integrate protective and vulnerability traits into more comprehensive risk profiles. Such broad integration may likely need to rely on data-driven approaches such as deterministic and learning-based clustering algorithms [54–56]. Given the availability of validated brief personality questionnaires [57] and the increasing implementation of online self-reports into routine data collection [58], systematic personality profiling in clinical settings should be considered. Having information on patients’ and clients’ constitutional strengths and vulnerability traits would likely help tailor care to their individual needs (e.g., when devising a suicide safety plan) [59], and could lead to performant AI-based suicide risk prediction models in the long run [60,61].

## 5. Conclusions

Our study highlights the clinical importance of considering personality when appraising suicide risk. Our results suggest that conscientiousness may be associated with self-destructive behavior in depressed middle-aged and older subpopulations despite its positive association with self-care in community samples. Even though highly conscientious middle-aged and older adults struggling with depression may appear clinically reassuring by being well organized, contained, and diligent, clinicians should remain vigilant and systematically assess depression severity and suicide risk in these individuals. The bulk of the behavioural patterns defining personality emerge in response to environmental factors encountered in childhood and early adulthood, providing no guarantee that they remain effective in situations faced only later in life, such as severe illness and functional decline, widowhood, empty nest syndrome (children moving out of home), end of professional growth, or the increasing awareness of one’s inevitable mortality. Future research should disentangle which of these specific aging-related challenges may increase suicide risk in more conscientious middle-aged and older depressed adults and for which ones conscientiousness may mitigate risk.

## Supplementary Material

supplement

Appendix A. Supplementary data

Supplementary data to this article can be found online at https://doi.org/10.1016/j.comppsych.2024.152523.

## Figures and Tables

**Table 1:** Sample characteristics.

	Total sample N = 313	Recent attempters *n* = 84	Non-recent attempters & Non-attempters *n* = 229	*p*-value for group differences

Sociodemographic variables				
Age in years	62.13 (7.55)	62.00 (8.47)	62.18 (7.21)	0.860
Female sex - count (%)	167 (53.35%)	43 (51.19%)	124 (54.15%)	0.736
Race - count (%):				0.253
White	265 (84.66%)	73 (86.90%)	192 (83.84%)	-
African-American	42 (13.42%)	8 (9.52%)	34 (14.85%)	-
Asian	4 (1.28%)	2 (2.38%)	2 (0.87%)	-
Multiple races	2 (0.64%)	1 (1.19%)	1 (0.44%)	-
Education in years	14.89 (2.81)	13.86 (2.76)	15.26 (2.74)	**<0.001**
Marital status - count (%):				0.216
Married/cohabitating	108 (34.62%)	24 (28.57%)	84 (36.84%)	-
Divorced/separated	103 (33.01%)	35 (41.67%)	68 (29.82%)	-
Never married	66 (21.15%)	15 (17.86%)	51 (22.37%)	-
Widowed	35 (11.22%)	10 (11.90%)	25 (10.96%)	-
Per capita yearly income in US$ - median (IQR)	20,500 (21,500)	20,500 (21,500)	20,500 (21,250)	0.660
Five-factor personality dimensions				
Conscientiousness mean item score	3.42 (0.71)	3.59 (0.74)	3.35 (0.69)	**0.012**
Neuroticism mean item score	3.30 (0.81)	3.23 (0.91)	3.32 (0.77)	0.434
Extraversion mean item score	2.86 (0.64)	2.99 (0.71)	2.82 (0.61)	**0.045**
Openness mean item score	3.18 (0.37)	3.10 (0.38)	3.21 (0.37)	**0.020**
Agreeableness mean item score	3.80 (0.38)	3.79 (0.38)	3.81 (0.39)	0.650
Health indicators				
HRSD total score* (depression severity)	19.17 (5.41)	19.88 (6.29)	18.90 (5.04)	0.203
DRS total score (cognitive functioning)	135.61 (5.12)	133.95 (5.80)	136.34 (4.72)	**0.001**
CIRS-G total score	8.94 (4.52)	8.61 (4.76)	9.06 (4.44)	0.452
Severe physical illness^ - count (%)	95 (30.35%)	35 (41.67%)	60 (26.20%)	**0.012**
Suicidal ideation and behavior characteristics				
Any current suicidal ideation - count (%)	227 (72.52%)	83 (98.81%)	144 (62.88%)	**<0.001**
SSI score - median (IQR)	13 (22)	26 (8.5)	5 (16)	**<0.001**
Any suicidal behavior in lifetime - count (%)	128 (40.89%)	84 (100%)	44 (19.21%)	**<0.001**
SIS score of most recent attempt	-	18.89 (5.24)	-	-
SIS score of most medically serious attempt	-	18.58 (5.44)	-	-
BLS score of most recent attempt	-	3.45 (2.11)	-	-
BLS score of most medically serious attempt	-	4.01 (2.04)	-	-
Age at the most recent attempt	-	61.83 (8.52)	-	-
Number of lifetime attempts - median (IQR)	-	1 (1.25)	-	-

Note: Presented statistics are means (standard deviation) unless otherwise specified. Recent attempters refer to participants with a suicide attempt within the past two years. Bolded *p*-values indicate significant group differences at *p* < 0.05.

IQR, interquartile range; HRSD, Hamilton Rating Scale for Depression; DRS, Mattis Dementia Rating Scale; CIRS-G, Cumulative Illness Rating Scale - Geriatrics; SSI, Beck Scale for Suicide Ideation; SIS, Beck Suicide Intent Scale; BLS, Beck Lethality Scale;

*, HRSD total computed without the suicide item (Item 3);

^, severe physical illness: a score of 3 or 4 on any organ system (domain) of the CIRS-G, excluding psychiatric illness.

**Table 2:** Summary table of logistic and linear regression models predicting presence (upper) and severity (lower) of recent suicidal behavior.

Single predictor models (Q1a-b)			Covariate-adjusted models (Q2a-b)		Interaction models (Q3a-b)	
(Q1-3a) Logistic regression predicting *presence of suicidal behavior* within the past two years (n = 313)						

Independent variables	OR (95%CI)	*p*-value	OR (95%CI)	*p*-value	OR (95%CI)	*p*-value

Conscientiousness	**1.41 (1.09, 1.84)**	**0.010**	**1.44 (1.09, 1.90)**	**0.010**	**1.86 (1.28, 2.69)**	**0.001**
Age	-		0.88 (0.67, 1.17)	0.386	0.92 (0.69, 1.22)	0.565
Female sex (ref. male)	-		0.97 (0.57, 1.65)	0.916	0.97 (0.57, 1.66)	0.912
Depression severity	-		1.17 (0.89, 1.52)	0.260	1.20 (0.91, 1.58)	0.191
Cognitive functioning	-		**0.68 (0.52, 0.89)**	**0.005**	**0.69 (0.53, 0.91)**	**0.010**
Severe physical illness (ref. none)	-		**1.91 (1.11, 3.30)**	**0.020**	**2.07 (1.19, 3.62)**	**0.011**
Conscientiousness* Depression severity	-		-		0.93 (0.70, 1.25)	0.647
Conscientiousness* Cognitive functioning	-		-		1.04 (0.78, 1.40)	0.766
Conscientiousness* Severe physical illness (ref. none)	-		-		**0.54 (0.30, 0.97)**	**0.039**

(Q1-3b) Linear regression predicting *intent severity* at the most recent attempt in participants with suicidal behavior within the past two years (n = 84)						

Independent variables	β estimate (SE)	*p*-value	β estimate (SE)	*p*-value	β estimate (SE)	*p*-value

Conscientiousness	**1.40 (0.58)**	**0.018**	**1.60 (0.62)**	**0.012**	1.35 (0.90)	0.140
Age	-		0.10 (0.69)	0.886	0.10 (0.68)	0.879
Female sex (ref. male)	-		−1.83 (1.20)	0.131	−1.85 (1.19)	0.124
Depression severity	-		1.02 (0.60)	0.093	0.91 (0.60)	0.130
Cognitive functioning	-		0.69 (0.73)	0.347	0.83 (0.72)	0.258
Severe physical illness (ref. none)	-		0.93 (1.21)	0.443	1.33 (1.21)	0.277
Conscientiousness* Depression severity	-		-		−1.18(0.67)	0.085
Conscientiousness* Cognitive functioning	-		-		0.32 (0.62)	0.609
Conscientiousness* Severe physical illness (ref. none)	-		-		1.17 (1.27)	0.360
Model statistics	F(1, 82) = 5.81, *p* = 0.018		F(6, 77) = 2.00, *p* = 0.076		F(9, 74) = 1.90, *p* = 0.065	
	R^2^ = 0.07		R^2^ = 0.14		R^2^ = 0.19	

Note: All continuous variables are mean-centered. Bolded test statistics indicate significance at *p* < 0.05. For the interpretation of the significant interaction term in Model Q3a, refer to Table 3.

**Table 3:** Simplified binomial logistic regression model decomposing the conscientiousness by severe physical illness interaction present in model Q3a.

Dependent variable: presence of suicidal behavior within the past two years (n = 313)			

Reference group	Independent variables	OR (95% CI)	*p*-value

*low* conscientiousness & *absence of* severe physical illness	*high* conscientiousness & *presence of* severe physical illness	**3.03 (1.38, 6.70)**	**0.006**
	*high* conscientiousness & *absence of* severe physical illness	**2.36 (1.23, 4.66)**	**0.011**
	*low* conscientiousness & *presence of* severe physical illness	**3.43 (1.57, 7.58)**	**0.002**

Note: Binomial logistic regression model including dummy variables for combinations of high-low conscientiousness combined with presence-absence of severe physical illness. The “low conscientiousness – absence of severe physical illness” variable serves as reference group and has been left out of the model. The cutoff for high vs low conscientiousness was determined by median split. Bolded test statistics indicate significance at *p* < 0.05.

## Data Availability

Data is available from the corresponding author upon reasonable request.
