# Supplementary material for: Diligent for better or worse: Conscientiousness is associated with higher likelihood of suicidal behavior and more severe suicidal intent in later life
Source: Compr Psychiatry. Author manuscript; Available in PMC 2025 Jul 17. (PMC12268956; doi:10.1016/j.comppsych.2024.152523)
Supplement: supplement [file NIHMS2095576-supplement-supplement.docx]

**Supplementary material to the article “Diligent for better or worse: conscientiousness is associated with higher likelihood of suicidal behavior and more severe suicidal intent in later life”**

**Figure S1** - Spearman correlations between all independent variables used in principal and sensitivity analyses

*Note: numbers indicate correlation coefficients; barred numbers are non-significant correlations.*

**Table S1 –** Sensitivity analysis testing robustness of the main effect of conscientiousness

|  | **Models adjusted for education** | | **Models adjusted for personality dimensions** | | **Models adjusted for education & personality dimensions** | |
| --- | --- | --- | --- | --- | --- | --- |
| Logistic regression predicting presence of suicidal behavior within the last two years (n = 313) | | | | | | |
| Independent variables | OR (95% CI) | p-value | OR (95% CI) | p-value | OR (95% CI) | p-value |
| Conscientiousness | **1.44 (1.10, 1.90)** | **.008** | **1.47 (1.08, 2.04)** | **.017** | **1.48 (1.08, 2.06)** | **.017** |
| Education in years | **0.58 (0.43, 0.76)** | **<.001** | **-** | | **0.62 (0.46, 0.83)** | **.002** |
| Neuroticism | - |  | 1.14 (0.83, 1.56) | .419 | 1.16 (0.84, 1.60) | .361 |
| Extraversion | - |  | 1.33 (0.98, 1.84) | .074 | 1.31 (0.96, 1.82) | .096 |
| Openness to experience | - |  | **0.66 (0.50, 0.86)** | **.003** | 0.76 (0.56, 1.01) | .062 |
| Agreeableness | - |  | 0.92 (0.71, 1.20) | .542 | 0.90 (0.69, 1.18) | .454 |
| Linear regression predicting intent severity at the most recent attempt in participants with suicidal behavior within the last two years (n = 84) | | | | | | |
| Independent variables | β estimate (SE) | p-value | β estimate (SE) | p-value | β estimate (SE) | p-value |
| Conscientiousness | **1.21 (0.55)** | **.031** | 1.03 (0.72) | .159 | 1.17 (0.73) | .113 |
| Education in years | 1.05 (0.55) | .061 | - | | 0.72 (0.60) | .236 |
| Neuroticism | - |  | -0.92 (0.74) | .218 | -0.72 (0.75) | .343 |
| Extraversion | - |  | -0.56 (0.69) | .419 | -0.48 (0.69) | .484 |
| Openness to experience | - |  | 0.60 (0.58) | .310 | 0.31 (0.63) | .623 |
| Agreeableness | - |  | **-1.35 (0.56)** | **.018** | **-1.27 (0.56)** | **.026** |
| Model statistics | F(2, 81) = 4.30, p = .017  R^2^ = 0.10 | | F(5, 78) = 2.71, p = .026  R^2^ = 0.15 | | F(6, 77) = 2.51, p = .029  R^2^ = 0.16 | |

*Note: all continuous variables are mean-centered. Effects of interest are highlighted in grey.*

**Table S2 –** Sensitivity analysis testing robustness of the conscientiousness-by-severe physical illness effect predicting presence of recent suicidal behavior

| Logistic regression predicting presence of suicidal behavior within the last two years (n = 313) | | | | | | | | | | | | | | | | | | | |  |
| --- | --- | --- | --- | --- | --- | --- | --- | --- | --- | --- | --- | --- | --- | --- | --- | --- | --- | --- | --- | --- |
|  | | **Model adjusted for years of education** | | **Model adjusted for neuroticism** | | | | **Model adjusted for extraversion** | | | | | **Model adjusted for openness to experience** | | | **Model adjusted for agreeableness** | | | |  |
| Independent variables | OR (95% CI) | | p-value | | OR (95% CI) | p-value | | | | OR (95% CI) | p-value | | | OR (95% CI) | p-value | OR (95% CI) | | p-value | |  |
| Conscientiousness | **1.84 (1.30, 2.69)** | | **.001** | | **1.71 (1.17, 2.55)** | | **.007** | | **1.59 (1.08, 2.39)** | | | **.021** | | **2.04 (1.42, 3.05)** | **<.001** | | **1.83 (1.29, 2.65)** | | **.001** | |
| Severe physical illness (vs. none) | **2.10 (1.18, 3.72)** | | **.011** | | **2.15 (1.24, 3.73)** | | **.006** | | **2.24 (1.30, 3.86)** | | | **.004** | | **2.22 (1.27, 3.88)** | **.005** | | **2.28 (1.31, 3.96)** | | **.003** | |
| Education in years | **0.60 (0.41, 0.84)** | | **.005** | | - | |  | | - | | |  | | - |  | | - | |  | |
| Neuroticism | - | |  | | 0.85 (0.58, 1.22) | | .377 | | - | | |  | | - |  | | - | |  | |
| Extraversion | - | |  | | - | |  | | 1.34 (0.93, 1.97) | | | .120 | | - |  | | - | |  | |
| Openness to experience | - | |  | | - | |  | | - | | |  | | **0.66 (0.46, 0.93)** | **.018** | | - | |  | |
| Agreeableness | - | |  | | - | |  | | - | | |  | | - |  | | 1.03 (0.75, 1.43) | | .844 | |
| Conscientiousness*Severe physical illness | **0.53 (0.30, 0.94)** | | **.030** | | 0.62 (0.33, 1.15) | | .129 | | 0.59 (0.32, 1.09) | | | .091 | | **0.49 (0.27, 0.88)** | **.018** | | **0.54 (0.30, 0.95)** | | **.032** | |
| Education* Severe physical illness | 0.99 (0.54, 1.77) | | .962 | | - | |  | | - | | |  | | - |  | | - | |  | |
| Neuroticism*Severe physical illness | - | |  | | 1.64 (0.92, 2.99) | | .099 | | - | | |  | | - |  | | - | |  | |
| Extraversion*Severe physical illness | - | |  | | - | |  | | 0.68 (0.37, 1.23) | | | .204 | | - |  | | - | |  | |
| Openness to experience*Severe physical illness | - | |  | | - | |  | | - | | |  | | 1.09 (0.63, 1.89) | .747 | | - | |  | |
| Agreeableness*Severe physical illness | - | |  | | - | |  | | - | | |  | | - |  | | **0.54 (0.29, 0.95)** | | **.037** | |

*Note: all continuous variables are mean-centered. The effect of interest is highlighted in grey.*
